# RNA-Seq Reveals a Role for NFAT-Signaling in Human Retinal Microvascular Endothelial Cells Treated with TNFα

**DOI:** 10.1371/journal.pone.0116941

**Published:** 2015-01-24

**Authors:** Sara R. Savage, Colin A. Bretz, John S. Penn

**Affiliations:** 1 Department of Pharmacology, Vanderbilt University School of Medicine, Nashville, Tennessee, United States of America; 2 Department of Cell and Developmental Biology, Vanderbilt University School of Medicine, Nashville, Tennessee, United States of America; 3 Department of Ophthalmology and Visual Sciences, Vanderbilt University School of Medicine, Nashville, Tennessee, United States of America; Queen’s University Belfast, UNITED KINGDOM

## Abstract

TNFα has been identified as playing an important role in pathologic complications associated with diabetic retinopathy and retinal inflammation, such as retinal leukostasis. However, the transcriptional effects of TNFα on retinal microvascular endothelial cells and the different signaling pathways involved are not yet fully understood. In the present study, RNA-seq was used to profile the transcriptome of human retinal microvascular endothelial cells (HRMEC) treated for 4 hours with TNFα in the presence or absence of the NFAT-specific inhibitor INCA-6, in order to gain insight into the specific effects of TNFα on RMEC and identify any involvement of NFAT signaling. Differential expression analysis revealed that TNFα treatment significantly upregulated the expression of 579 genes when compared to vehicle-treated controls, and subsequent pathway analysis revealed a TNFα-induced enrichment of transcripts associated with cytokine-cytokine receptor interactions, cell adhesion molecules, and leukocyte transendothelial migration. Differential expression analysis comparing TNFα-treated cells to those co-treated with INCA-6 revealed 10 genes whose expression was significantly reduced by the NFAT inhibitor, including those encoding the proteins VCAM1 and CX3CL1 and cytokines CXCL10 and CXCL11. This study identifies the transcriptional effects of TNFα on HRMEC, highlighting its involvement in multiple pathways that contribute to retinal leukostasis, and identifying a previously unknown role for NFAT-signaling downstream of TNFα.

## Introduction

Diabetic retinopathy (DR) is one of the leading causes of irreversible vision loss in the US, blinding approximately 12% of diabetic patients every year.[1,2] Inflammation is an important component of DR, with a number of cytokines and adhesion proteins induced by or increased in the diabetic milieu that play significant roles in diabetes-induced retinal pathology.[3] Tumor necrosis factor-alpha (TNFα) is one such soluble pro-inflammatory cytokine, and multiple reports have observed increased vitreous levels in patients with DR.[4–6] In particular, TNFα is implicated as a contributing factor in the development of retinal leukostasis, with both pharmacologic blockade and genetic deletion of TNFα having been shown to inhibit leukostasis in diabetic rodents.[7,8] Leukostasis is the firm adherence of myeloid-derived cells to the endothelium, and is a common pathogenic feature of DR often associated with chronic retinal inflammation. Increased numbers of adherent leukocytes are observed in the retinas of DR patients, where they co-localize with dead or injured endothelial cells.[9,10] Adherent leukocytes can further damage the retinal endothelium by secreting proteolytic enzymes and/or occluding retinal capillaries, ultimately leading to focal ischemia and apoptosis of cells associated with the capillary unit.[11,12] Focal ischemia causes the surrounding tissue to become hypoxic and increases the production of vasoactive factors that promote pathologic neovascularization, which is considered to be a defining feature of late stage DR.[3] These findings indicate an important role for TNFα in the overall pathology of retinal leukostasis and progression of retinopathy; but the transcriptional effects of TNFα on retinal microvascular endothelial cells (RMEC) are not completely understood.

The nuclear factor of activated T-cell (NFAT) signaling pathway is one of many activated by TNFα, and numerous TNFα-induced inflammatory proteins are also known NFAT family gene targets, though to date no studies have identified a role for NFAT signaling in the context of TNFα-treated retinal vascular endothelium.[13–19] NFAT is a family of five proteins grouped for their similarity to Rel/NF-κB family transcription factors. NFATc denotes the four isoforms (NFATc1, NFATc2, NFATc3, and NFATc4) regulated by the serine phosphatase calcineurin (CN).[20,21] CN activates NFATc proteins through its binding to a conserved Ca^2+^/CN-dependent translocation regulatory domain, and this association can be effectively disrupted using the small organic molecule Inhibitor of NFAT-calcineurin Association-6 (INCA-6), which competitively binds to the discrete NFAT binding site of CN, blocking NFAT activity without altering CN phosphatase activity.[22,23]

In the present study, we investigated the transcriptional effect of TNFα on human retinal microvascular endothelial cells (HRMEC), and whether NFAT signaling contributes to this response, by performing RNA-seq analysis on primary HRMEC treated with TNFα in both the presence and absence of the NFAT-specific inhibitor INCA-6. These data characterize the role of TNFα-induced inflammation on HRMEC and give insight into new therapeutic targets for DR.

## Materials and Methods

### RMEC cell culture

Primary HRMEC (catalog #ACBRI 181) were purchased from Cell Systems (Kirkland, WA) and were cultured in flasks coated with attachment factor (Cell Signaling; Danvers, MA). Growth medium consisted of endothelial basal medium (EBM; Lonza; Walkersville, MD) supplemented with 10% FBS and endothelial cell growth supplements (EGM SingleQuots; Lonza). All cultures were incubated at 37°C, in 5% CO_2_ and 95% relative humidity. Passage 3 cells were used for these experiments.

### Treatment and RNA isolation

HRMEC were cultured to near confluence in 6-well dishes coated with attachment factor, before being serum starved (0.5% FBS in EBM) for 12 hrs. Cells were then treated with 1 ng/ml TNFα (Sigma-Aldrich; St. Louis, MO) in the presence or absence of 1.0 μM INCA-6 (Tocris; Minneapolis, MN). After 4 hrs of treatment, cells were lysed and RNA purified using a Qiagen RNeasy kit (Qiagen; Valencia, CA) in accordance with the manufacturer’s protocol.

### Library preparation and sequencing

Total RNA samples were submitted to the Vanderbilt VANTAGE core for sequencing. RNA sample quality was confirmed using the 2100 Bioanalyzer (Agilent Technologies; Santa Clara, CA). All RNA samples had an RNA integrity number > 9.0. Samples were prepared for sequencing using the TruSeq RNA Sample Prep Kit (Illumina; San Diego, CA) to enrich for mRNA and prepare cDNA libraries. Library quality was assessed using the 2100 Bioanalyzer. Sequencing was performed using a single read, 50 bp protocol on the Illumina HiSeq 2500 (Illumina). The sequence data can be found at the NCBI Short Read Archive with accession number SRP047271.

### RNA-seq alignment and differential expression

Sequence alignment and differential expression analyses were expedited using the Vanderbilt VANGARD core. Alignment to the UCSC human reference genome hg19 was performed using TopHat v2.0.9 with default parameters.[24] Mapped reads were then analyzed for differential expression using MultiRankSeq, which utilizes DESeq, edgeR, and baySeq algorithms.[25] Briefly, MultiRankSeq uses raw read counts to first cluster samples according to gene expression profiles to assure sample homogeneity within treatment groups. The read counts are then used to determine differential expression by DESeq, edgeR, and baySeq. An overall ranking of a gene is determined by the sum of its rankings from all three methods. Comparisons were made between the TNFα-treated group and the control group, and between the TNFα group and the TNFα plus INCA-6 group. Transcripts were filtered to those having a false discovery rate (FDR) < 0.05 in all three methods.

### Pathway analysis

The Database for Annotation, Visualization and Integrated Discovery (DAVID) v6.7 was used for pathway enrichment analysis.[26,27] Lists of differentially expressed genes were submitted to the DAVID website and compared to a background of human reference genes. Pathway enrichment was determined using the Kyoto Encyclopedia of Genes and Genomes (KEGG) Pathway annotation. Pathways were considered significantly enriched with p < 0.05.

### qRT-PCR validation of RNA-seq results

cDNA was reverse transcribed using the High-Capacity cDNA Archive Kit (Applied Biosystems; Carlsbad, CA) according to the manufacturer’s instructions. Quantitative real-time RT-PCR was performed by co-amplification of the gene of interest (*CXCL10*, *CXCL11*, *SELE*, *ICAM1*, or *VCAM1*) vs. *β-actin* (endogenous normalization control), using gene-specific TaqMan Gene Expression Assays (Applied Biosystems). Expression data were analyzed using the comparative Ct method and significance determined using a student’s T-test for each targeted gene. Analysis was done not only on the samples submitted for RNA-seq analysis, but also on samples from additional biologically-independent experimental replicates.

## Results

### RNA-seq quality and alignment

We performed RNA-seq using 3 samples each of HRMEC treated with vehicle, TNFα in vehicle, or TNFα in vehicle with INCA-6. Total reads varied between 24,038,972 to 35,171,982 among the 9 samples over a total of 33,240 unique transcripts (**Table 1**). There was no statistical difference between the number of reads in each treatment group (ANOVA, p = 0.21). Before mapping to the reference genome, 2,119 to 5,365 reads were removed due to low quality. On average, 97% of the transcripts mapped to the UCSC human genome hg19.

**Table 1:** Summary of reads mapping to the human genome (UCSC hg19) using Tophat v2.0.9.

	Control				TNFα				TNFα + INCA-6			
	1	2	3	Avg	1	2	3	Avg	1	2	3	Avg
Total Reads	36,407,423	39,019,063	33,043,496	36,156,661	43,028,634	34,303,038	38,356,120	38,562,597	28,981,471	36,874,690	29,505,055	31,787,072
Reads Removed	2328	5365	2161	3285	3089	2701	3006	2932	2085	2724	2119	2309
% Mapped	96.1%	97.8%	96.0%	97.0%	96.6%	98.1%	97.1%	97.0%	97.9%	97.9%	97.9%	98.0%

### Differential expression

**Figure 1:**
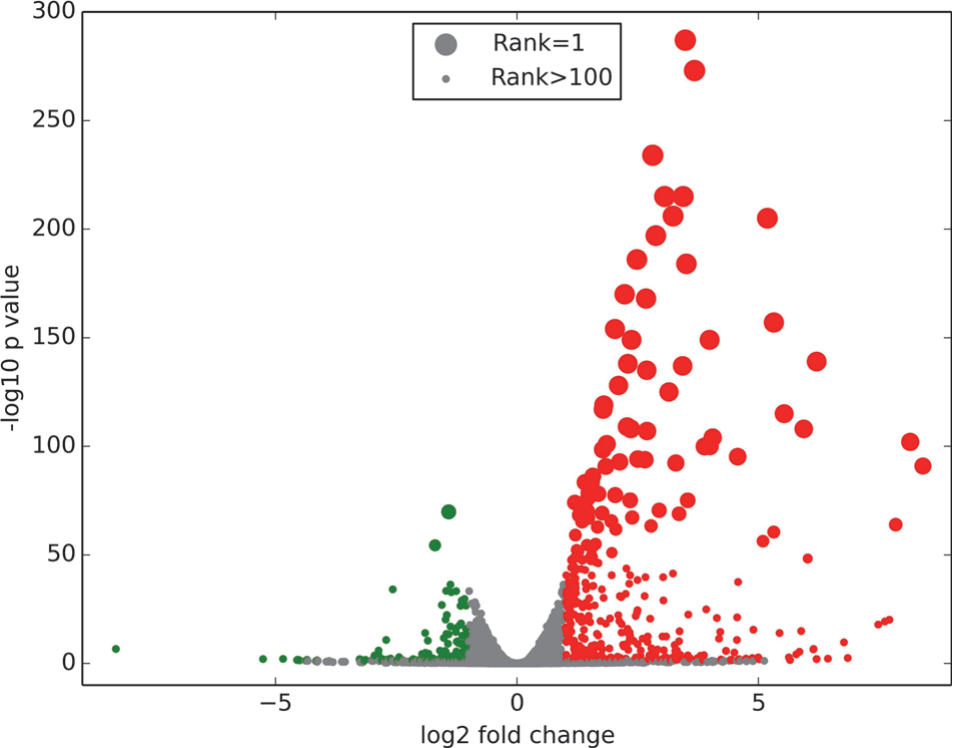
Volcano plot of the fold change of transcripts in TNFα-treated HRMEC compared to control using edgeR. Red circles indicate upregulated genes while green circles indicate downregulated genes. Circle size indicates gene rank using MultiRankSeq.

Differential expression was determined using three different algorithms: DESeq, edgeR, and baySeq. Comparisons were made between the TNFα-treated HRMEC and the control cells, and between the TNFα with INCA-6 and the TNFα-treated cells. We narrowed the list of transcripts to those considered significantly changed (FDR < 0.05) by all three algorithms. The data is summarized in **Table 2**. Compared to control, TNFα treatment changed expression of 744 genes, primarily by upregulation (**Fig. 1**). Of the 744 genes that were differentially expressed, 579 were upregulated, and over 50% of those were upregulated by more than 2 fold (**S1 Table**). Only 18 genes were differentially expressed in the TNFα with INCA-6 group compared to cells treated with TNFα alone.

**Table 2:** Summary of RNA-seq differential expression analysis.

	Transcripts with FDR < 0.05	Upregulated Transcripts	Downregulated Transcripts
TNFα vs Control	744	579	165
TNFα + INCA-6 vs TNFα	18	5	13

### TNFα effect on HRMEC gene expression

The top 10 genes upregulated and downregulated in HRMEC by TNFα are summarized in **Table 3**. The products of several of these genes have well characterized roles in leukostasis. Notably *VCAM1*, *ICAM1*, and *CXCL10*, genes known for their roles in vascular adhesion, were three of the highest expressed genes in the TNFα-treated samples. The gene with the lowest expression was *KCNK2*, a potassium channel that negatively regulates leukocyte transmigration.[28]

**Table 3:** Top 10 upregulated and downregulated genes by TNFα in HRMEC.

Ensembl Gene ID	Gene Symbol	log2FoldChange	p Value	FDR
**ENSG00000162692**	VCAM1	9.05161	<0.00001	<0.00001
**ENSG00000169245**	CXCL10	8.40542	1.83E-94	1.17E-91
**ENSG00000049249**	TNFRSF9	8.14305	1.04E-105	8.28E-103
**ENSG00000237988**	OR2I1P	7.84193	3.18E-67	1.37E-64
**ENSG00000173391**	OLR1	7.7114	5.57E-23	7.71E-21
**ENSG00000213886**	UBD	7.6186	3.41E-22	4.51E-20
**ENSG00000267607**	CTD-2369P2.8	7.48056	1.05E-20	1.30E-18
**ENSG00000023445**	BIRC3	7.10132	<0.00001	<0.00001
**ENSG00000235947**	EGOT	6.77046	2.96E-12	2.03E-10
**ENSG00000090339**	ICAM1	6.66984	<0.00001	<0.00001
**ENSG00000250961**	CTD-2023N9.1	−1.84767	3.59E-06	0.000115
**ENSG00000107562**	CXCL12	−1.89446	3.26E-07	1.26E-05
**ENSG00000171227**	TMEM37	−1.90168	1.14E-16	1.12E-14
**ENSG00000226808**	LINC00840	−1.96633	1.36E-06	4.79E-05
**ENSG00000164089**	ETNPPL	−2.44162	5.27E-05	0.001298
**ENSG00000003137**	CYP26B1	−2.57184	3.28E-37	7.89E-35
**ENSG00000162009**	SSTR5	−2.70851	2.12E-13	1.63E-11
**ENSG00000187513**	GJA4	−2.8654	2.62E-08	1.21E-06
**ENSG00000232259**	RP11-4C20.3	−2.86794	5.51E-06	0.000172
**ENSG00000082482**	KCNK2	−2.94959	7.59E-06	0.00023

Fold changes and p-values reported were calculated by the edgeR algorithm.

**Figure 2:**
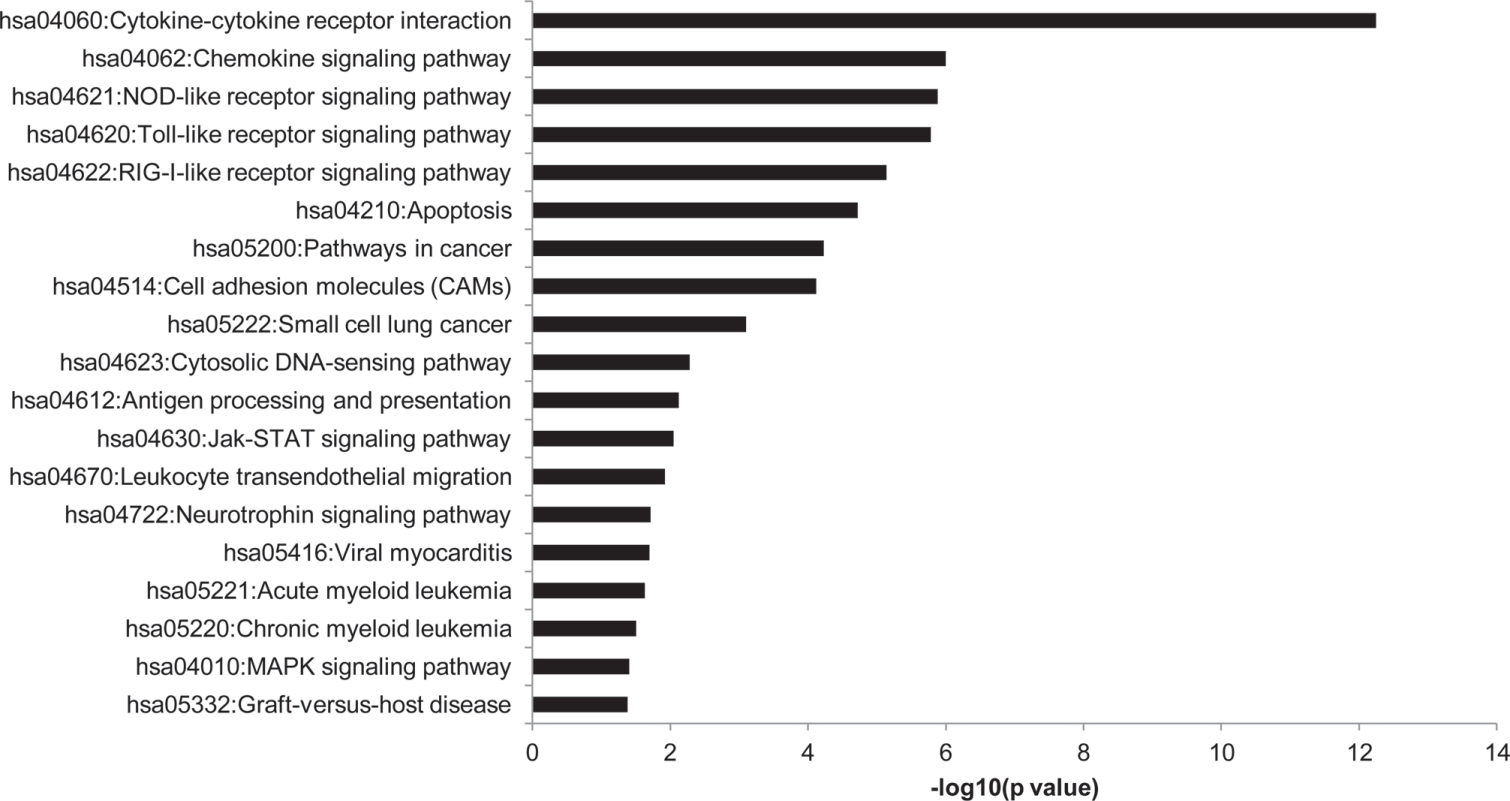
KEGG pathway enrichment in TNFα-treated HRMEC. Pathway enrichment was determined using DAVID and a p value < 0.05.

To further characterize the differentially expressed genes in TNFα-treated HRMEC, we used the KEGG database to determine pathway enrichment, with results shown in **Fig. 2**. According to the KEGG database, 19 pathways were enriched. Among these pathways are several that are particularly related to our research, including cytokine-cytokine receptor interaction (44 transcripts), cell adhesion molecules (19 transcripts), and leukocyte transendothelial migration (13 transcripts). As expected, the pathway analysis also highlighted the role of TNFα in both MAPK (21 transcripts) and chemokine signaling (27 transcripts).

### INCA-6 effect on TNFα-treated HRMEC

INCA-6 changed the expression of 18 genes compared to HRMEC treated with TNFα alone. Of these 18 genes, 13 were also differentially expressed in TNFα-treated cells compared to control. INCA-6 exacerbated the effects of TNFα on three of these genes (*FRY*, *TNIP3*, *SQSTM1*), and INCA-6 counteracted the upregulated expression of the other 10 genes that had been affected by TNFα (**Table 4**).

**Table 4:** Genes that were significantly upregulated by TNFα and downregulated by INCA-6.

**Ensembl Gene ID**	Gene Symbol	log2FoldChange	p Value	FDR
**ENSG00000006210**	CX3CL1	−1.17657	1.64E-19	7.43E-16
**ENSG00000169248**	CXCL11	−0.76461	3.10E-14	1.09E-10
**ENSG00000227507**	LTB	−0.7322	1.65E-07	0.000145
**ENSG00000102934**	PLLP	−0.71586	8.91E-09	1.01E-05
**ENSG00000162692**	VCAM1	−0.62413	4.59E-22	2.43E-18
**ENSG00000121858**	TNFSF10	−0.61602	4.93E-11	1.20E-07
**ENSG00000146374**	RSPO3	−0.59714	5.70E-11	1.29E-07
**ENSG00000188015**	S100A3	−0.58063	8.14E-07	0.000549
**ENSG00000143387**	CTSK	−0.54792	8.57E-08	7.99E-05
**ENSG00000124875**	CXCL6	−0.46089	2.21E-07	0.000184

**Figure 3:**
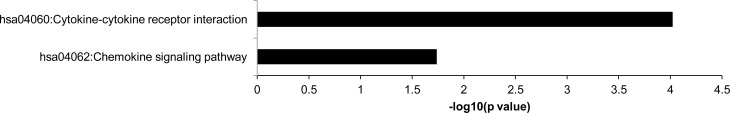
KEGG pathways enriched by INCA-6 treatment in HRMEC. Pathway enrichment was determined using DAVID and a p value < 0.05.

KEGG pathway enrichment analysis shown in **Fig. 3** revealed half of these genes to play a role in cytokine-cytokine receptor interaction (*TNFSF10*, *CXCL6*, *CX3CL1*, *CXCL11*, *LTB*). Notably, *VCAM1* upregulation by TNFα was also counteracted by INCA-6.

### Validation of five differentially regulated genes by qRT- PCR

**Figure 4:**
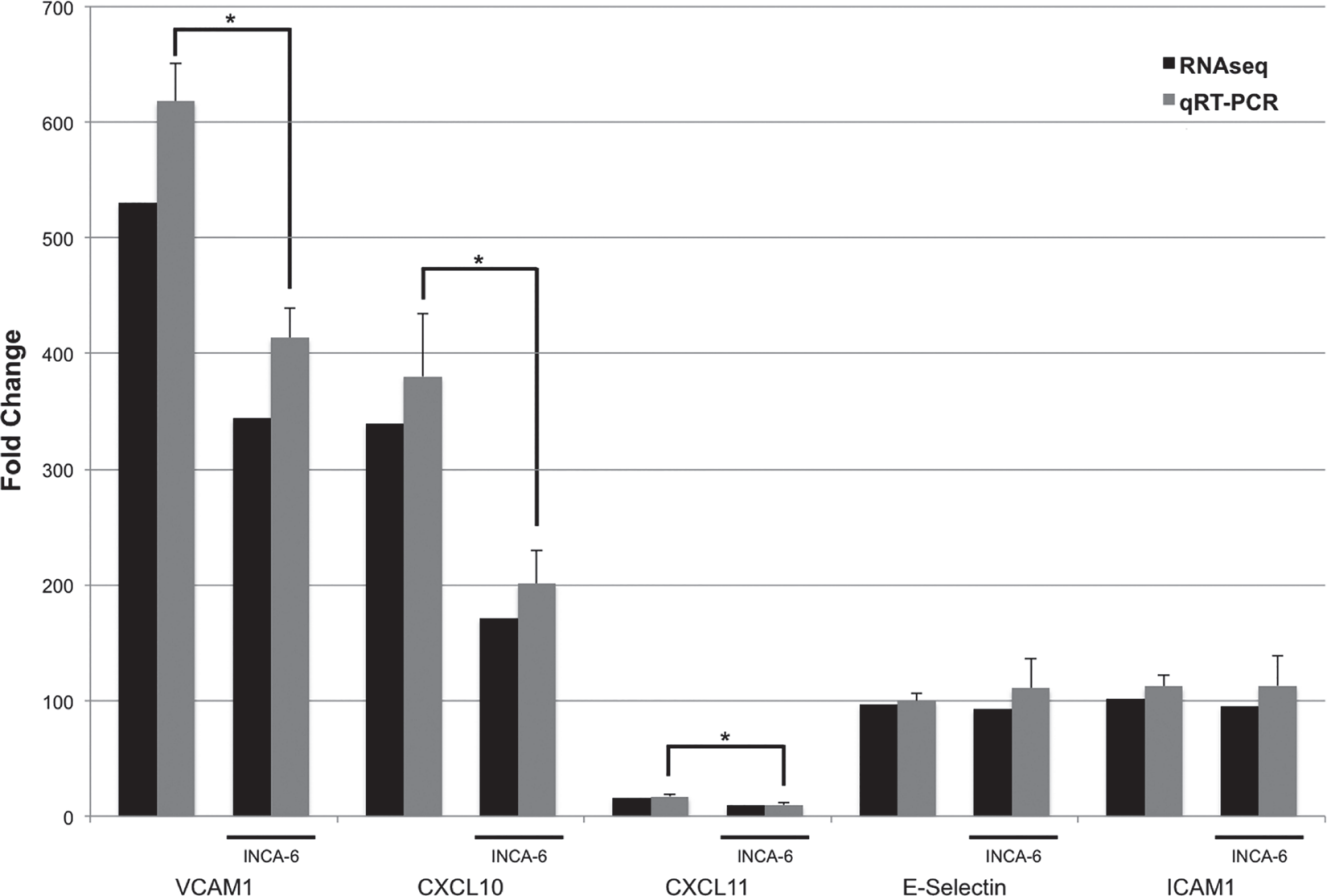
qRT-PCR validation of several differentially expressed genes from the RNA-seq data. Black bars indicate fold change from the RNA-seq data calculated by edgeR. Fold change for qRT-PCR (gray bars) was determined by the relative Ct method normalized to β-actin (*p<0.001).

In order to confirm the findings from the RNA-seq, we chose to validate five different genes by performing qRT-PCR on the sequenced samples as well as samples from a second biologically independent experiment (**Fig. 4**). qRT-PCR analysis showed that TNFα treatment caused upregulation of *CXCL10*, *CXCL11*, *SELE*, *ICAM1*, and *VCAM1* in HRMEC (p < 0.0001), and INCA-6 significantly reduced expression of *CXCL10*, *CXCL11*, and *VCAM1*, but not *SELE* or *ICAM1* compared to TNFα-treated cells (p < 0.0001). This qRT-PCR data is consistent with the RNA-seq findings, showing similar patterns for both TNFα-induced changes and the effect of NFAT inhibition.

## Discussion

This study provides a characterization of the effect of TNFα on retinal microvascular endothelial cells. Furthermore, it elucidates a role for NFAT signaling in mediating the effect of TNFα on RMEC. RNA-seq analysis revealed that TNFα stimulated the differential expression of a number of genes, particularly those related to cytokine-cytokine receptor interaction, cell adhesion, and leukocyte transendothelial migration. Three of the genes most highly upregulated by TNFα were *ICAM1*, *VCAM1*, and *SELE*, which code for adhesion proteins ICAM1, VCAM1, and E-Selectin. These proteins are known to be regulated by TNFα and have been shown to mediate the effect of TNFα on leukocyte adhesion on other endothelial cell types.[29,30] Genes coding for the cytokines CCL2, CXCL6, CXCL10, CXCL11, and IL-8 were also all notably upregulated by TNFα, and these proteins play well-defined roles in the recruitment of leukocytes to inflamed or damaged endothelium.[31–34] Additionally, the gene with the largest reduction in expression by TNFα was *KCNK2*, which encodes the TWIK-related potassium channel-1 (TREK1). Blockade of TREK1 channel activity or reduced expression of *KCNK2* has been shown to increase leukocyte transmigration across brain endothelial cells.[28] Altogether, these changes in gene expression support TNFα as an inflammatory factor in RMEC and a contributor to retinal leukostasis.

In addition to characterizing the effect of TNFα on RMEC, this study also provides the first insight into how NFAT family transcription factors modulate TNFα signaling in the retinal endothelium. TNFα is known to activate NFAT signaling in macrophages, and a number of studies have shown a role for NFAT-induced TNFα expression, but to date none have looked at a role for NFAT downstream of TNFα in endothelial cells.[13,35,36] Our study found that INCA-6, a specific NFAT inhibitor, reduced expression of a small subset of genes that were upregulated by TNFα. Interestingly, this subset included the previously discussed *VCAM1*, *CXCL6*, and *CXCL11*, as well as *CX3CL1* and *TNFSF10*. CX3CL1 is an inflammatory cytokine that, in its soluble form, assists in recruitment of leukocytes to areas of inflammation and in its membrane-bound form aids in leukocyte tethering and adhesion, while *TNFSF10* is the gene encoding TNF-related apoptosis-inducing ligand (TRAIL), a cytokine that promotes endothelial cell apoptosis in addition to leukocyte adhesion.[37,38] Apoptotic death of endothelium is a well recognized and critical feature of diabetic retinopathy.[39] Of note, the qRT-PCR data shown in **Fig. 4** also shows that *CXCL10* expression is reduced with INCA-6 treatment. This effect is significant in our qRT-PCR data; however *CXCL10* is not included in the **Table 4** gene list, as only two of the three analyses (DESeq and edgeR) reported it as significantly altered by INCA-6 treatment. These data support a role for NFAT in TNFα-induced inflammation.

It is important to note that our data may be biased towards highly expressed transcripts and that sequencing at a greater depth may elucidate additional targets and pathways affected by INCA-6. However, these data present a good path forward for dissecting the role of NFAT in retinal inflammation and leukostasis.

Taken together, these findings suggest that TNFα regulates leukostasis at least partially through NFAT signaling. As TNFα has an important role in retinal inflammation and DR, NFAT may represent an attractive target for therapeutics aimed at retinal leukostasis in DR. Future studies will focus on the role of individual NFAT-isoforms in the context of TNFα-induced leukostasis, as inhibition of critical isoforms may allow for tuning of therapeutic strategies aimed at specific disease related processes, while allowing beneficial NFAT signaling to continue.

## Supporting Information

S1 Table List of differentially expressed genes in TNFα-treated HRMEC compared to control. (XLSX)
